# Slip versus Slop: A Head-to-Head Comparison of UV-Protective Clothing to Sunscreen

**DOI:** 10.3390/cancers14030542

**Published:** 2022-01-21

**Authors:** Elizabeth G. Berry, Joshua Bezecny, Michael Acton, Taylor P. Sulmonetti, David M. Anderson, Haskell W. Beckham, Rebecca A. Durr, Takahiro Chiba, Jennifer Beem, Douglas E. Brash, Rajan Kulkarni, Pamela B. Cassidy, Sancy A. Leachman

**Affiliations:** 1 Department of Dermatology, Oregon Health & Science University, Portland, OR 97239, USA; kulkarnr@ohsu.edu (R.K.); cassidyp@ohsu.edu (P.B.C.); leachmas@ohsu.edu (S.A.L.); 2 College of Osteopathic Medicine of the Pacific, Western University of Health Sciences, Lebanon, OR 97355, USA; joshua.bezecny@westernu.edu; 3 Exponent, Inc., Natick, MA 01760, USA; macton@exponent.com; 4 Exponent, Inc., Atlanta, GA 30326, USA; tsulmonetti@exponent.com (T.P.S.); danderson@exponent.com (D.M.A.); 5 Columbia Sportswear Company, Portland, OR 97229, USA; HBeckham@columbia.com (H.W.B.); rdurr@mit.edu (R.A.D.); taka.chiba@columbia.com (T.C.); Jennifer.Beem@columbia.com (J.B.); 6 Departments of Therapeutic Radiology and Dermatology, Yale University, New Haven, CT 06520, USA; douglas.brash@yale.edu; 7 Portland Veterans Administration Medical Center, Portland, OR 97239, USA

**Keywords:** photoprotective clothing, photoprotection, sun protection factor (*SPF*), ultraviolet protection factor (*UPF*), critical wavelength (CW), skin cancer, melanoma

## Abstract

**Simple Summary:**

Photoprotection reduces invasive melanoma incidence and mortality, but not all sun protection modalities are created equal. Dermatologists have long debated the pros and cons of photoprotective clothing and sunscreen, but few studies compare the effectiveness of these two modalities head-to-head. This study uses both in vitro and in vivo techniques to compare the ultraviolet radiation (UVR) protective capacity of four modern textiles and two commercially available, broad-spectrum sunscreens.

**Abstract:**

Ultraviolet radiation (UVR) exposure is the most important modifiable risk factor for skin cancer development. Although sunscreen and sun-protective clothing are essential tools to minimize UVR exposure, few studies have compared the two modalities head-to-head. This study evaluates the UV-protective capacity of four modern, sun-protective textiles and two broad-spectrum, organic sunscreens (*SPF* 30 and 50). Sun Protection Factor (*SPF*), Ultraviolet Protection Factor (*UPF*), Critical Wavelength (CW), and % *UVA*- and % UVB-blocking were measured for each fabric. *UPF*, CW, % *UVA*- and % UVB-blocking were measured for each sunscreen at 2 mg/cm^2^ (recommended areal density) and 1 mg/cm^2^ (simulating real-world consumer application). The four textiles provided superior UVR protection when compared to the two sunscreens tested. All fabrics blocked erythemogenic UVR better than the sunscreens, as measured by *SPF*, *UPF*, and % UVB-blocking. Each fabric was superior to the sunscreens in blocking full-spectrum UVR, as measured by CW and % *UVA*-blocking. Our data demonstrate the limitations of sunscreen and UV-protective clothing labeling and suggest the combination of *SPF* or *UPF* with % *UVA*-blocking may provide more suitable measures for broad-spectrum protection. While sunscreen remains an important photoprotective modality (especially for sites where clothing is impractical), these data suggest that clothing should be considered the cornerstone of UV protection.

## 1. Introduction

### 1.1. UVB and UVA Cause Skin Cancer via Cyclobutane Pyrimidine Dimers (CPDs)

Exposure to ultraviolet radiation (UVR) remains the most important and most modifiable risk factor for the development of skin cancer [1,2]. As skin cancer incidence rises worldwide, minimization of exposure to UVR is critical for reducing the morbidity, mortality, and cost associated with skin cancer [3]. To achieve this, the American Academy of Dermatology recommends sun avoidance, application of broad-spectrum sunscreen (*SPF* 30 or higher), and use of hats and protective clothing [4]. Since avoidance of UVR is not always possible, sunscreen and sun-protective clothing are essential features of a multi-pronged approach to skin cancer prevention. Recent data from Queensland, Australia underscore the fact that photoprotection is effective. Aitken et al. showed a decline in invasive melanoma incidence and mortality in individuals under age 40 years in Queensland [5]. The steepest decline was in those born after 1980, the decade in which photoprotection education initiatives such as the “Slip” (on a shirt), “Slop” (on sunscreen), and “Slap” (on a hat) campaign were implemented [5,6].

Both UVB (280–315 nm) and *UVA* (315–400 nm) radiation are carcinogenic. Of note, UVC (100–280 nm) is the most damaging type of UVR but is completely attenuated by ozone in the atmosphere before reaching the earth’s surface. The UV portion of solar radiation that reaches the earth’s surface comprises 5% UVB and 95% *UVA* radiation. UVB is a higher energy radiation but is less able to penetrate through the skin than the longer wavelengths of *UVA*. UVB causes erythema of skin and has historically been implicated as the main contributor to the development of skin cancer [7]. UVB is directly absorbed by DNA bases resulting in the formation of cyclobutane pyrimidine dimers (CPDs) and 6-4 photoproducts that can result in mutations if not repaired [7]. *UVA* causes pigment darkening or tanning of the skin and is associated with photoaging [8]. Recent work shows that *UVA* has a much greater role than previously understood in skin cancer development due to its ability to induce reactive oxygen and nitrogen species, and ultimately generate CPDs hours after *UVA* exposure [9,10,11,12]. 

UV-induced CPDs are more mutagenic than 6-4 photoproducts, likely due to their slower repair [13]. The most common UV-induced mutation in human skin cancer is the C>T transition. This mutation can be caused by the rapid deamination of cytosine (C) or 5-methylcytosine in CPDs, transforming them into uracil and thymidine, respectively. Error-free replication of the deaminated CPDs by DNA polymerase η then passes on C>T mutations to daughter cells [14]. The CPDs thereby cause skin cancer [15].

### 1.2. Sunscreens and Clothing Protect against UVR-Induced Mutagenesis 

Active ingredients of sunscreens fall into two categories: inorganic (also known as mineral or physical) and organic (also referred to as chemical). Inorganic ingredients include zinc oxide (ZnO) and titanium dioxide (TiO_2_). New data suggest that these inorganic compounds primarily absorb UV radiation within the UVB and short *UVA* wavelengths and reflect radiation in the long *UVA* and visible wavelengths [16]. Organic UVR filters contain aromatic hydrocarbons that absorb photons in the UV spectrum and emit lower-energy, longer wavelength photons and/or heat that do not damage the skin [17]. Both inorganic and organic sunscreens prevent actinic keratoses (premalignant keratinocytic neoplasms) and squamous cell carcinoma [18,19,20]. Sunscreen is also effective in prevention of basal cell carcinoma and melanoma [18,21,22]. Furthermore, routine sunscreen application prevents photoaging [17].

Clothing provides protection by scattering and absorbing UVR. Mouse models have shown that sun-protective clothing can prevent skin cancer, but little human data exist in the literature [23]. However, since UVR is a causal agent in skin cancer, protection with clothing is likely to be dependent on the degree of protection provided from UVR. The degree of protection depends on the color, material, fiber, yarn and fabric structure [24]. Fabric structure is one of the most important factors, with the least porous material providing the greatest protection [25,26,27]. Synthetic fabrics have demonstrated the highest UV protection [28,29,30]. Dark colors absorb more UVR and thus provide higher protection than light colors [29,31,32,33]. Other factors that impact penetration of UVR through the clothing include: stretch, wetness, wear (from use or washing), color loss (bleaching), UVR-absorbing additives, and yarn morphology [34,35,36,37]. Although all clothing blocks some degree of UVR, some studies suggest many commonly worn fabrics may provide insufficient UVR protection [29,38,39].

### 1.3. Measures of UVR Protection

***The sun protection factor (SPF)*** rating traditionally used in sunscreen labeling is measured by exposing small areas of skin of human subjects (Fitzpatrick skin types I–III) to simulated solar radiation for varying durations. The smallest dose of UVR that produces visible, well-circumscribed redness on tested skin is called the minimal erythemal dose (*MED*). *SPF* is the ratio of the *MED* with sunscreen uniformly applied at 2 mg/cm^2^ (*MED_protected_*) to that of skin without sunscreen (*MED_unprotected_*):

(1) $$SPF=\frac{MED_{protected}}{MED_{unprotected}}\;$$

Simply stated, *SPF* reflects the factor by which one can spend more time in the sun without getting burned. For example, *SPF* 30 would theoretically allow someone who would normally burn in 10 min to be exposed for 300 min before burning, assuming constant incident solar UVR energy. 

**Figure 1:**
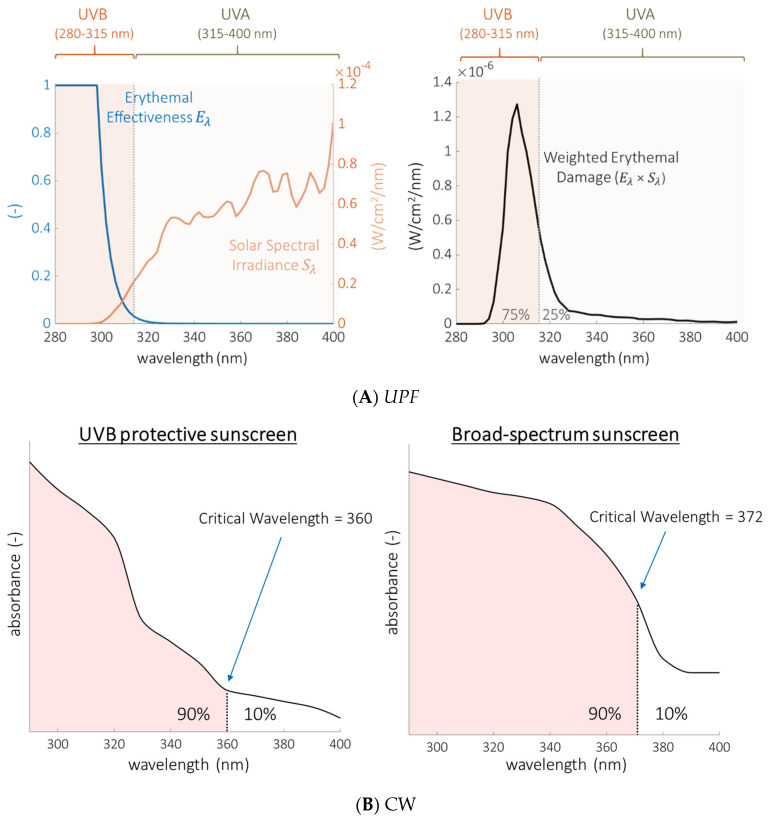
Ultraviolet protection factor (*UPF*) is primarily a measure of UVB protection whereas critical wavelength (CW) is a measure of the degree of broad-spectrum protection. (**A**) *UPF* is a mathematical function designed to recapitulate sun protection factor (*SPF*) from a laboratory measurement of transmittance and is weighted toward the UVB portion of the spectrum. Left: Plots of the erythemal effectiveness function (*E_λ_*) and the solar spectral irradiance (*S_λ_*) over the UVR spectral range. Right: The product of *E_λ_* and *S_λ_* has a peak that lies predominantly (75%) within the UVB range (280–315 nm). Very little of the *UPF* function comes from wavelengths longer than 360 nm, where the UVR intensity is highest but the erythemal effectiveness is near zero [48]. (**B**) Visual representation of the CW as the wavelength below which 90% of the total absorbance (area under the curve) in the UVR region is contained. Left: Hypothetical sunscreen that primarily blocks UVB radiation has a critical wavelength below the 370 nm threshold required by the FDA to be labeled broad-spectrum. Right: Hypothetical sunscreen with improved *UVA* blocking performance meets the broad-spectrum criterion. A sunscreen could meet the CW criterion of 370 nm without comprehensively blocking *UVA* radiation.

***The ultraviolet protection factor (UPF)*** rating traditionally used for clothing labeling requires a laboratory spectrophotometer to measure UVR transmittance (the fraction of UVR that is transmitted through the clothing). Although related, *SPF* and *UPF* are not equivalent [40]. In contrast to *SPF* measurements (that utilize UVB-induced erythema as the readout), *UPF* objectively measures all wavelengths of solar simulated light transmitted through the clothing and then applies weighting constants to mathematically recapitulate *SPF* testing. These constants (known as the erythemal effectiveness function and solar spectral irradiance) heavily weight *UPF* toward the UVB wavelengths (Figure 1A) because these are the primary wavelengths that induce erythema in the skin. The *UPF* of a garment represents how much erythemally-weighted UVR is transmitted through the clothing. *UPF* values are the ratio of erythemally-weighted UVR detected with or without a specimen (clothing) between the light source and detector:

(2) $$UPF=\frac{\sum_{280\mathrm{nm}}^{400\mathrm{nm}}E_{\lambda }\times S_{\lambda }\times \Delta \lambda }{\sum_{280\mathrm{nm}}^{400\mathrm{nm}}E_{\lambda }\times S_{\lambda }\times T_{\lambda }\times \Delta \lambda }$$

*E_λ_* is the relative erythemal spectral effectiveness, a constant that adjusts for the ability of each wavelength (*λ*) to generate cutaneous erythema [41]. *S_λ_* is the solar spectral irradiance (Wm^−2^ nm^−1^), a constant that represents sun intensity at each wavelength at noon in Albuquerque, New Mexico [42]. *T_λ_* is the average measured spectral transmittance of the specimen and Δ*λ* is the measured wavelength interval (nm). 

Both of these measures represent the combined effect of incident dose and a biological response. This biological response, slight reddening, is related to discomfort but it is blistering sunburn that is related to skin cancer [43,44]. Neither *SPF* or *UPF* rating systems take into consideration the fact that non-erythema-inducing UVR wavelengths are carcinogenic. Nor do they take into consideration the fact that erythema and skin cancer have different dose-dependencies. One measure currently used to address this deficit in evaluating the performance of sunscreen is the critical wavelength. 

***The critical wavelength (CW, λ_c_)*** is calculated from the measured absorbance of a sunscreen across the entire UV spectrum and is intended to provide an objective quantification of how well a sunscreen reduces exposure to both UVB and *UVA* wavelengths [45]. Absorbance (*A*), is the negative of the (base ten) logarithm of transmittance (*T*):

(3) $$A\left(\lambda \right)=-\log_{10}\left[T\left(\lambda \right)\right]$$

For example, at a given wavelength, a sunscreen or garment that blocks 90% of incident UV radiation has an absorbance of 1 [−log (0.1)], while one that blocks 99% of incident UV has an absorbance of 2 [−log (0.01)]. The transmittance of a sunscreen or textile is measured with a spectrophotometer equipped with an integrating sphere, which allows capture of all radiation that is diffusely scattered and transmitted through the sample. It is important to note that absorbance, as used in the quantitative expressions described here, represents more than just absorbed light; it also includes alternate means of radiation attenuation including the reflection and/or scattering of incident light.

The critical wavelength is the wavelength below which 90% of the total absorbance (*A*) of a sunscreen in the atmosphere-penetrating UVR region is contained (Figure 1B):

(4) $$\int_{290\mathrm{nm}}^{\lambda_{c}}A\left(\lambda \right)d\lambda =0.9\int_{290\mathrm{nm}}^{400\mathrm{nm}}A\left(\lambda \right)d\lambda$$

In other words, the absorbance of a particular sunscreen is measured as a function of wavelength between 290–400 nm (UVB from 290–315 and *UVA* from 315–400). UVB-specific sunscreen ingredients are not effective at preventing transmission in the *UVA* range, producing absorbance curves that peak at the shorter end of the wavelength spectrum. In contrast, *UVA* filters shift the absorbance curves to the right at the longer *UVA* wavelengths. Sunscreens that block both *UVA* and UVB radiation have absorbance curves that extend across the majority of the spectral region (Figure 1B). The area under the curve is summed (integrated) from 290 nm to 400 nm. The critical wavelength is the wavelength at which 90% of the total area under the absorbance curve is reached. Therefore, a higher critical wavelength indicates that the absorbance of a material is proportionally greater at longer wavelengths and offers more relative protection from *UVA* radiation. However, a *UVA*-specific agent with little absorbance in the UVB range could have a very high critical wavelength yet inadequate protection against UVB or sunburn. Therefore, the critical wavelength should never be interpreted in isolation and is only meaningful when *SPF* is also taken into consideration. The United States Food and Drug Administration (FDA) requires sunscreen to have a CW greater than 370 nm to be labeled as broad-spectrum [46]. 

***% UV-Blocking.*** The critical-wavelength requirement does not exist for garments. Instead, some countries require sun-protective garments to have less than 5% transmittance in the *UVA* region (T(*UVA*), Equation (5)) [47].

(5) $$T\left(UVA\right)=\frac{\sum_{315\mathrm{nm}}^{400\mathrm{nm}}T_{\lambda }\times \Delta \lambda }{\sum_{315\mathrm{nm}}^{400\mathrm{nm}}\Delta \lambda }$$

These values are often reported as % *UVA* blocking = 100% − *T*(*UVA*), where *T*(*UVA*) is expressed as a percentage. Similarly, transmittance in the UVB region, T(UVB), is determined using an analogous equation but evaluating wavelengths from 280 to 315 nm, and % UVB blocking = 100% − T(UVB) where T(UVB) is expressed as a percentage.

In this paper, we compare the *SPF*, *UPF*, CW, and % UV-Blocking of commercial sunscreens to those of modern sun-protective clothing. We follow this with a discussion of the consequences of exposure of human skin to *UVA* radiation and summarize new data on the mutagenic properties of visible light. We highlight the potential of sun-protective clothing to offer superior protection from the underappreciated risks posed by these wavelengths of solar radiation.

## 2. Materials and Methods

### 2.1. Fabrics

**Figure 2:**
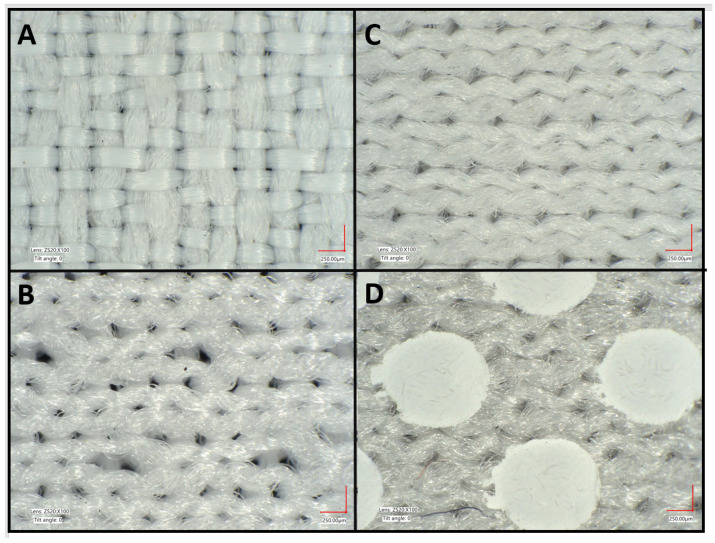
Four commercial fabrics were selected for study: (**A**) 107-gsm (g/m^2^) nylon woven, (**B**) 180-gsm polyester pique knit, (**C**) 90-gsm polyester interlock knit, and (**D**) 95-gsm polyester interlock knit with TiO_2_ dot print at 30% surface coverage. These fabrics are currently used in sun-protective apparel. Each fabric is constructed of multifilament synthetic yarns from 50 to 160 denier. Fabric images were taken using a Keyence VHX-7000 Digital Microscope (Itasca, IL, USA). Scale bar is 250 µm.

Four fabric samples (Table 1, Figure 2) used in commercial sun-protective apparel (Columbia Sportswear, Portland, OR, USA) were chosen for the study because they are representative of modern sun-protective materials: one nylon woven and three polyester knit fabrics. The three knit fabrics include two different common knit structures, a pique and an interlock. Of the two interlock knit fabrics, one includes a TiO_2_ dot print covering about 30% of its surface. These white dots are present for heat mitigation, and work by reflecting more solar radiation and emitting more thermal radiation than the underlying polyester fabric [49]. These four fabrics are generally thinner and lighter than the ones for which *UPF* and *SPF* data have been previously reported in the literature [24]. All fabrics were dyed to be off-white.

### 2.2. Sunscreens

Two organic, broad-spectrum sunscreens (*SPF* 30 and *SPF* 50, Table 1) were selected for comparison with the four fabrics. The sunscreens were the same brand and contained identical active ingredients: avobenzone, homosalate, octisalate, and octocrylene. The *SPF* 50 sunscreen had a higher percentage of homosalate (10% vs. 8%) and octocrylene (8% vs. 6%). These active ingredients are four of the sixteen UV filters listed in the FDA Code of Federal Regulations [50] and are a common combination used in multiple brands of U.S. sunscreens. Tests were performed with newly opened, unexpired products.

### 2.3. In Vitro UPF Testing

#### 2.3.1. Fabric *UPF*

*UPF* measurements of the textile fabrics were conducted as described in the American Association of Textile Chemists and Colorists (AATCC) Test Method 183 [48]. Briefly, each dry, unstretched fabric was placed in a UV-2000S Ultraviolet Transmittance Analyzer (Labsphere Inc., North Sutton, NH, USA) to capture diffuse transmittance from 280 to 400 nm. Five unique measurements were taken at different sample orientations rotated by 45° between each measurement. *UPF* was calculated for the transmission spectrum of each sample using the formula in Equation (2), and then an average *UPF* and standard deviation were calculated from the individual *UPF* values [48].

#### 2.3.2. Sunscreen *UPF*

*UPF* measurements of two commercial broad-spectrum sunscreens were conducted using AATCC Test Method 183 [48]. Of note, many studies refer to spectrophotometric testing of sunscreens as “in vitro *SPF*” and to erythema measurements of textiles on human skin as “in vivo *UPF*.” To eliminate confusion, we will use the terms “sunscreen *UPF*” and “fabric *SPF*” throughout. The two sunscreens were applied in accordance with International Organization for Standardization (ISO) 2444 to UVR-transparent quartz slides at a density of 2 mg/cm^2^. Additional testing was performed at a density of 1 mg/cm^2^ to simulate real-world consumer application [51]. Transmittance was measured using a Thermo Fisher Evolution Bio260 spectrophotometer (Thermo Fisher Scientific, Waltham, MA, USA). To account for non-uniformity in coating thickness, three slides were prepared for each sunscreen *SPF* level and coating density. Ten measurements were taken in random positions on each slide. *UPF* was calculated for each measurement, and the *UPF* is reported as the mean and standard deviation of these measurements.

### 2.4. In Vivo SPF Testing

#### 2.4.1. *SPF* of Fabrics

The fabric *SPF* on human skin of the four textile samples were measured by a third party (AMA Laboratories, New City, NY, USA) using the standard procedure described by the International *SPF* Test Method of COLlPA, CTFA, JCIA and CTFA-SA [52]. The study was approved by the AMA Laboratories Internal Review Board. Briefly, the dose of UVR was supplied by either a 150 or 300 watt Xenon Arc Solar Simulator (Solar Light Co., Philadelphia, PA, USA), each with a continuous emission spectrum from 290 to 400 nm. Both were equipped with dichroic mirrors and a 1-mm Schott WG-320 filter to produce a simulated solar *UVA*-UVB emission spectrum. These were also equipped with a 1-mm UG 11 filter to remove reflected heat as well as visible and infrared radiation. 

A total of five healthy adult volunteers (ages 36 to 64 years) were selected by AMA laboratories. Three volunteers (one from each Fitzpatrick Skin Type I, II, and III to maximize the human variation studied) underwent *SPF* testing for each fabric [53] (Tables S1 and S2). All individuals provided written informed consent. The bilateral infrascapular area of each subject’s back was used as the test site. Test sites were cleaned with a dry cotton pad, and rectangular areas of at least 30 cm^2^ were demarcated. A minimum of five progressive UVR doses were administered within this site to determine a subject’s minimal erythemal dose on unprotected skin (*MED_unprotected_*). Each subject’s *MED_unprotected_* was defined as the shortest time of exposure (or lowest UVR dose required) that produced minimally perceptible erythema at 16 to 24 h post exposure. A control sunscreen with standard *SPF* 15 or 16 was used as a verification technical control. 

Once the *MED_unprotected_* had been determined, subjects returned to the lab for *SPF* testing of the fabrics. The test fabric was secured closely to each subject’s skin without stretching and using a thin layer of adhesive tape on the sample periphery to cover a minimum area of 30 cm^2^. Based upon each subject’s previously determine *MED_unprotected_*, the test areas were irradiated with a series of progressively higher UVR doses (minimum of five). The subjects returned to the testing facility 16–24 h after UVR exposure for determination of the MED of protected skin (*MED_protected_*) by a blinded evaluator. Each fabric was tested on three subjects and the mean *SPF* was determined for each individual fabric as the ratio of *MED_protected_*_/_*MED_unprotected_* (Equation (1)). 

#### 2.4.2. *SPF* of Sunscreens

For the purposes of this study, the package label *SPF*s of the commercial sunscreens were used. *SPF*s of commercial sunscreens in the U.S. are determined in accordance with FDA regulations as described in the Code of Federal Regulations 21 [46].

## 3. Results

### 3.1. In Vivo SPF Values Are Higher for Fabrics Than Sunscreens

In vivo *SPF* measurements of the fabrics ranged from 60 to 80, with the nylon woven fabric having the lowest *SPF* and the polyester interlock knit having the highest *SPF* (Table 2). All fabrics had a higher *SPF* than the *SPF*s on the sunscreen package labels. 

### 3.2. In Vitro UPF Values Are Higher for Fabrics Than Sunscreens

In vitro *UPF* measurements for all four fabrics exceeded 200 (Table 2). *UPF* values of sunscreens demonstrated dose- and *SPF*-dependence, as expected. Sunscreen B (*SPF* 50) applied at an areal density of 2 mg/cm^2^ showed the greatest *UPF* (31 ± 19), while Sunscreen A (*SPF* 30) applied at 1 mg/cm^2^ had the lowest *UPF* (5.3 ± 3.2). 

### 3.3. All Fabrics Have CWs That Meet the Criteria for Broad-Spectrum Labeling

All four fabrics had critical wavelengths (CWs) greater than or equal to 370 nm, the minimum value that the FDA requires for a sunscreen to be considered broad-spectrum (Table 2). Both sunscreens exhibited a broad-spectrum CW of at least 370 nm at an areal density of 2 mg/cm^2^, but not at 1 mg/cm^2^.

### 3.4. % UVB-Blocking Is Greater in Fabrics Relative to Sunscreens

**Figure 3:**
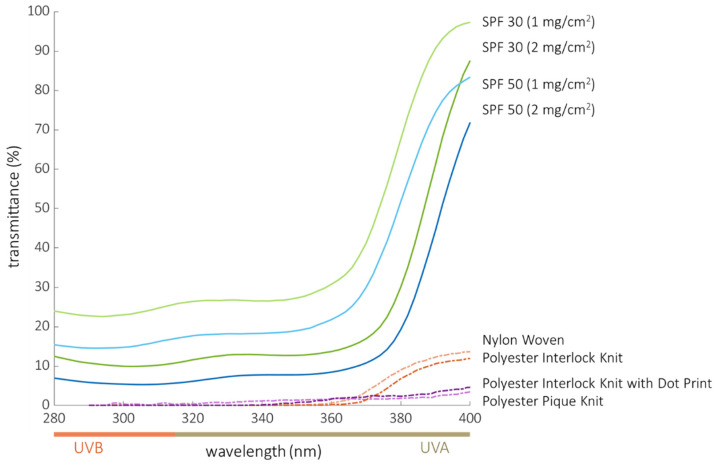
Transmittance curves of the two commercial sunscreens applied at 2 mg/cm^2^ and 1 mg/cm^2^ compared to curves of the four fabrics tested. Each curve is an average of multiple measurements.

All four textiles blocked > 99% of UVB (which equates to a transmittance < 1% in the UVB region) with little difference detected between the four fabric types (Table 2). Both sunscreens blocked UVB in a dose- and *SPF*-dependent manner (range of sunscreen protection = 76–94%), with neither providing as much protection as the fabrics. The fabrics exhibited consistently higher and less variable UVB blocking compared to the two commercial sunscreens at both areal densities. The UVR spectra of the textiles and sunscreens (Figure 3) show that all textiles had lower transmittance than the sunscreens over the UVB wavelengths.

### 3.5. % UVA-Blocking Is Greater in Fabrics Relative to Sunscreens

*UVA* blocking by the fabrics ranged from 96% to 98%, with the polyester fabrics having slightly better performance than the nylon fabric (transmittance < 4% in the *UVA* region, Table 2). As with UVB, sunscreen *UVA* blocking increased with areal density and *SPF*, ranging from 54% for the *SPF* 30 formulation applied at 1 mg/cm^2^ to 82% for the *SPF* 50 sunscreen applied at 2 mg/cm^2^. The best sunscreen *UVA* blocker, *SPF* 50 applied at 2 mg/cm^2^, did not block *UVA* as well as the lowest performing fabric (nylon, at 96%). All four textiles had much lower *UVA* transmittance than the sunscreens. The transmittance of all tested sunscreens increased rapidly in the *UVA* region from 380 to 400 nm (Figure 3).

## 4. Discussion

The four textiles in our study provided superior UVR protection when compared to the two commercial sunscreens tested (Table 2, Figure 3). All four fabrics successfully blocked erythemogenic UVR at a level better than the sunscreens, as measured by UVB transmittance, *SPF*, and *UPF* metrics. All four fabrics were superior to the sunscreens with respect to blocking full spectrum UVR, as measured by *UVA* transmittance and CW metrics. There was substantial disparity between the *SPF* and *UPF* values determined for both fabric and sunscreen. Despite previous reports to the contrary [40], this is not surprising given the fact that they are measured in different ways, using transmittance of UVR to calculate *UPF* in contrast to induction of a downstream cutaneous reaction (erythema) to calculate *SPF*. *UPF* is indirectly calculated using a mathematic weighting to simulate the erythemogenic potential of the UVR, whereas *SPF* directly measures development of erythema in response to UVR. In addition to the absolute performance of fabrics and sunscreens, there was substantial variability of protection by sunscreens in our study, despite great efforts made to assure consistency (Table 2). This variability is likely due to the difficulty in uniformly applying sunscreen and highlights an inherent weakness in sunscreens with respect to inconsistent application and the propensity of sunscreens to wear or wash off. Despite these challenges, sunscreens remain an important tool of our armamentarium against skin cancer when protective clothing is not an option. 

### 4.1. Tailored Use of Fabrics and Sunscreens

In general, utilization of both photoprotective clothing and sunscreen offers improved UV protection. However, some activities and climates make wearing photoprotective clothing difficult or undesirable, and it is impractical to cover the entire body with clothing. Similarly, sunscreens can be messy and difficult to apply and re-apply in proper amounts, especially after vigorous exercise or water-related activities. Ideally, these two photoprotective methods can be combined and tailored for each person and for the outdoor activities in which they are engaged. However, there are additional factors that should be considered with respect to the use of clothing and sunscreen. 

### 4.2. Limitations in the Use of Sunscreen for Photoprotection

**Figure 4:**
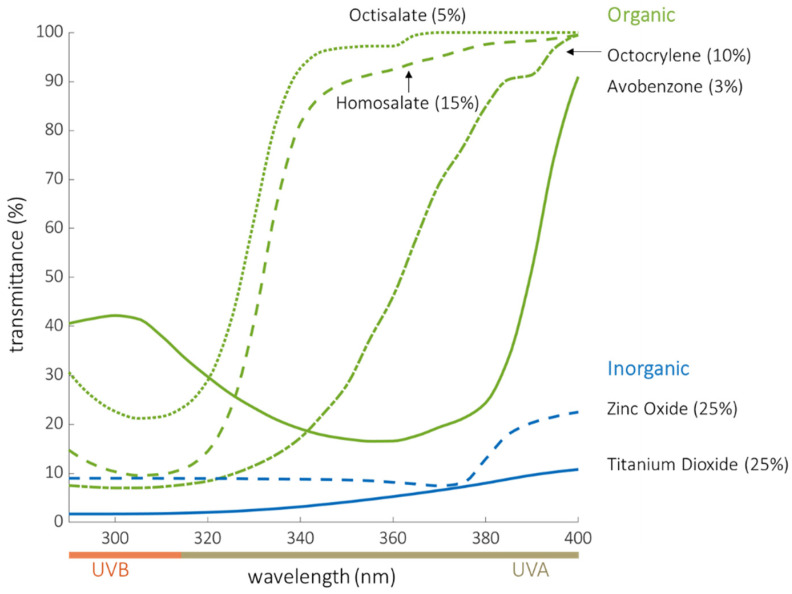
Transmittance of organic sunscreen filters (tested in this study) and inorganic sunscreen filters (ZnO and TiO_2_). Data were obtained from the BASF sunscreen simulator [59] where the maximum concentration allowable by the FDA [46] was used to generate each curve.

We now know that radiation across the entire UVR spectrum damages DNA. A review of the published absorbance spectra of active organic and inorganic sunscreens shows that nearly all U.S. sunscreen ingredients provide little or limited protection at wavelengths longer than 380 nm [54,55,56,57,58,59] (Figure 4). The curves in Figure 4 reflect the transmission of sunscreen ingredients at the maximum concentration allowed by the FDA. When applied at high concentrations (25%), the inorganic compounds (ZnO and TiO_2_) do provide superior protection across the UV spectrum compared to organic filters, but still show up to 20% transmittance beyond 380 nm. However, very few sunscreens contain inorganic filters at these high concentrations as they can be difficult to apply and have a chalky, cosmetically less acceptable appearance. Most inorganic sunscreens on the market contain 10–20% ZnO and 2–14% TiO2 [60]. It is also worth noting that eight broad-spectrum, organic UV filters commonly used in other countries but not yet approved by the FDA also show an increase in transmittance beyond 380 nm [61,62].

This gap in UVR protection is significant since long-wavelength *UVA* is capable of producing reactive oxygen species and CPDs that can lead to skin cancer [9,10,11]. Although the carcinogenic potential of UVB and UVC were first to be shown to produce mutagenic CPDs and 6-4 photoproducts in DNA, a robust body of literature now exists supporting the carcinogenic potential of *UVA* as well [9,10,11,63]. Recent studies also show that longer *UVA* wavelengths (*UVA*1, 340–400 nm) induce more solar UV-signature mutations than shorter UV wavelengths [64]. Lawrence and colleagues demonstrated that human skin irradiated at 385 nm generated CPDs, which increased for 2 h and persisted for 24 h without evidence of repair [65]. Additionally, irradiation of human skin with *UVA*1 and visible light produces biomarkers of DNA damage in the form of increased TP53 and BCL-2 expression, eliciting a DNA damage response without producing visible erythema [63]. Further, data from Runger et al. suggest that *UVA*-induced CPDs may be more mutagenic than those produced by UVB due to a lower and shorter-lived activation of protective cell cycle arrest pathways [66]. 

The high absorbance of textiles in the near *UVA* region raises the question of whether there are biological effects of visible light against which textiles might also be protective. UVR and visible light have been shown to generate reactive oxygen species (ROS) that damage sensitive biomolecules in the skin. ROS produced by solar radiation in the UVB, *UVA*, and visible wavelengths cause damage to DNA that is potentially mutagenic. Photons in the UV region are the most efficient at producing ROS, but studies of the action spectrum of sunlight indicate that more than 50% of free radicals, including ROS, arise from visible light with wavelengths in the range of 400 to 700 nm [67]. The free radicals produced by visible light have the same carcinogenic and ageing effects as their counterparts produced by UV. The principal differences in the effects of various wavelengths of light on generation of ROS in the skin are the chromophores that mediate radical production. Light-absorbing chromophores in the skin include nucleic acids, aromatic amino acids, urocanic acid, NADH and NADPH, cytochromes, riboflavins, porphyrins, melanin and its precursors, and β-carotene [68]. These chromophores can act as photosensitizers that catalyze the generation of ROS and reactive nitrogen species that, if not quenched by cellular antioxidant defenses, can damage sensitive biomolecules such as DNA, RNA, proteins and lipids. 

Blue light (400–500 nm) is known to generate pigmentary changes in human skin, particularly visible in persons with darker skin (Fitzpatrick phototypes III and IV). Irradiation of the skin on the backs of healthy volunteers with 415 nm blue-violet light produced hyperpigmentation, independent of p53 activation, that persisted for months after exposure [69]. In subsequent mechanistic studies, the laboratory of Thierry Passeron showed evidence that this effect is mediated by a dedicated sensor, opsin-3 (OPN3) which is expressed in melanocytes [70]. Activation of melanogenesis downstream of OPN3 is calcium dependent, activates the protein kinase CAMKII, and leads to the phosphorylation of the transcription factor MITF and thus increased transcription of melanin synthesis enzymes tyrosinase and dopachrome tautomerase (DCT). Blue light also facilitates the formation of a protein complex that contains tyrosinase and DCT. This complex leads to sustained tyrosinase activity that likely mediates the long-lasting hyperpigmentation that is observed in skin type III and higher.

Taken together, these data suggest that available sunscreens fail to protect against wavelengths of radiation, both UVB and *UVA*, that produce CPD and 6-4 photoproducts, which can result in mutations that drive carcinogenesis. In addition, there are hyperpigmentation effects of visible light, also not blocked by current sunscreens, that are of significant cosmetic concern to many individuals with darker skin. This makes protection from the full spectrum of UV and visible radiation an important goal, but one that may be difficult to achieve because of the strong cosmetic preference for transparent sunscreens. Sunscreens that protect in the 380–700 nm region are opaque to the human eye, so most sunscreens lack protection in the long *UVA* and visible spectrum. Importantly, photoprotective clothing may represent a partial solution to this problem since opaque clothing effectively blocks both UV and visible radiation. 

### 4.3. Limitations in the Use of Clothing for Photoprotection

**Figure 5:**
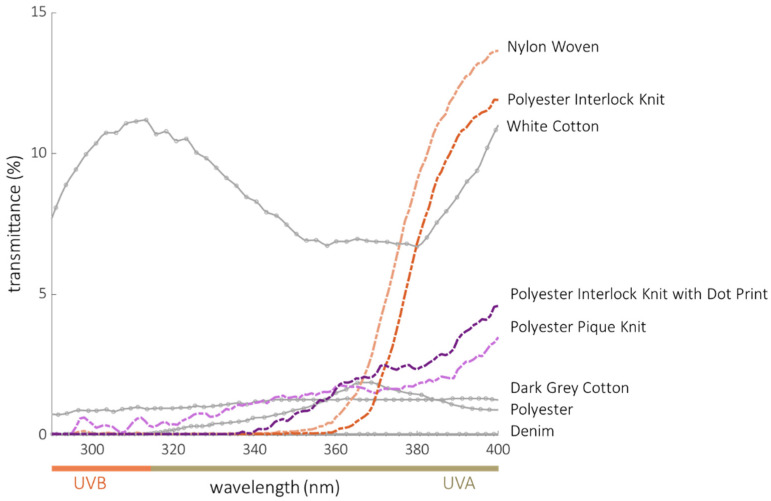
Transmittance spectra of the four fabrics designed for UV protection tested in this study (orange and purple lines) compared to “normal” clothing items tested by Coyne, et al. (gray lines). The data from Coyne, et al. were digitized, converted to transmittance and represent the average of the 16 measurements as reported in the original study. White cotton and dark grey cotton were GAP, Inc. 100% cotton shirts. The denim was 69% cotton, 30% polyester, 1% spandex GAP jeans. Polyester refers to a 84% polyester, 16% spandex Coolibar rash guard [71].

Few studies in the literature have directly compared the performance of UV-protective clothing to sunscreen. In 2018, Coyne et al. reported the broad-spectrum protection afforded by then-available commercial clothing [71]. Selected results from analysis of those data are shown in Figure 5 re-plotted as transmittance versus wavelength for comparison with the four fabrics that we tested. Because this figure only includes fabrics, the upper limit of the transmittance scale is 15%. In contrast, the transmittance scale in Figure 3, which includes both fabrics and sunscreens, must extend to 100% to display the increasing transmittance, and reduced protection, of the sunscreens beginning around 370 nm.

The Coyne data plotted in Figure 5 are summarized in Table 3. The denim jeans, dark grey cotton shirt, and polyester/spandex photoprotective rash guard provided the most protection. With the exception of the white cotton shirt, all fabrics blocked > 99% of UVB, blocked > 96% of *UVA*, and met the minimum critical wavelength (370 nm) to be considered broad-spectrum protection. The fabrics in our study provide comparable broad-spectrum protection (Figure 3, Table 2) without requiring dark dyes. Since lighter colored fabrics (such as those tested in our study) generally provide the least protection for a given fabric structure, dyes and pigments added for deeper coloration would lend even more UVR protection than that indicated in Table 2. As shown in the Coyne data, a common white cotton shirt does not typically provide as much UVR protection (*UPF* = 9, Table 3) as a dark grey shirt made of the same material (*UPF* = 98, Table 3). On the other hand, the denim tested in the Coyne study provided even better UVR protection (*UPF* = 2000, Table 3). While fabric weights and thicknesses were not provided in the Coyne study, denim is typically heavier and darker than fabrics used for shirts. Thus, the Coyne data additionally demonstrate that sufficiently heavy and dark fabrics, such as denim, will effectively block UVR. The primary challenge for textile engineers is to provide outstanding UVR protection in garments using fabrics that are lightweight and air permeable (attributes that are linked to comfort in hot environments), thereby increasing the likelihood that a person would choose clothing for photoprotection.

### 4.4. Limitations of Current Photoprotection Metrics

Our data and that of Coyne, et al. also demonstrate that CW alone does not provide a useful characterization of UVR protection. The white cotton fabric exhibits a critical wavelength of 389 nm (Table 3), which technically meets the FDA definition of broad-spectrum protection for a sunscreen. However, the same fabric has a *UPF* rating of 9 and the actual protection provided across wavelengths is the lowest of all of the fabrics in this comparison. The % *UVA* and UVB blocking values, 91.7% and 89.9%, respectively, are better indicators of the minimal level of broad-spectrum protection provided by this white cotton shirt.

**Figure 6:**
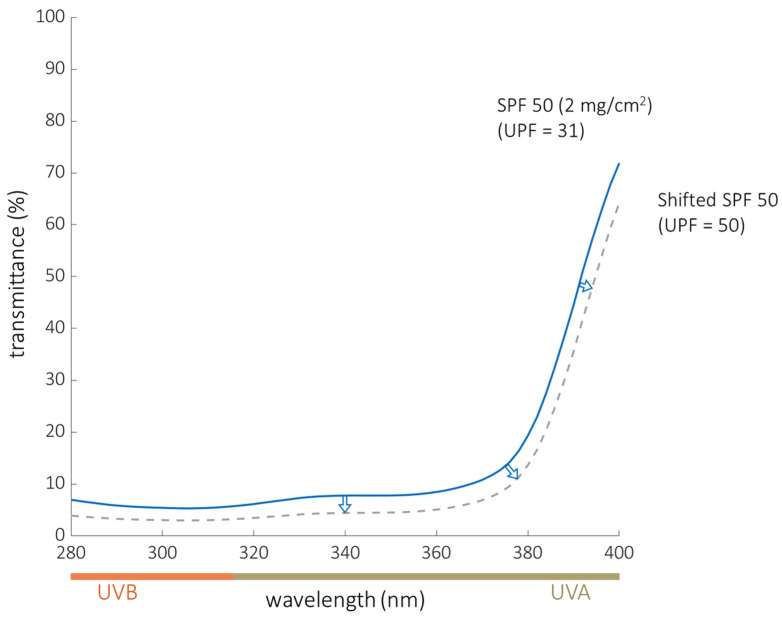
Illustration of the limitations of current measures of sun protection. The solid curve shows the measured transmittance of Sunscreen B applied at 2 mg/cm^2^, which has a *SPF* rating of 50 but had a measured *UPF* of only 31. The dashed curve was generated using a shifting function to simulate a transmittance curve with an average *UPF* of 50. For both the measured and simulated curve, the rapid increase in transmittance in the *UVA* region represents reduced protection from *UVA* radiation.

Additionally, the specific shape of the UV transmittance curve can generate misleading results for measurements of *SPF*, *UPF* and CW. In the case of materials that exhibit UV transmission that increases rapidly through the *UVA* region, it is possible for a sunscreen or a garment to provide poor UV protection despite having a seemingly adequate *SPF*, *UPF* and CW. We illustrate this concept in Figure 6 and Table 4. The solid curve represents the measured transmittance of Sunscreen B applied at 2 mg/cm^2^, which has a labeled *SPF* of 50 but a measured *UPF* of 31. The dashed curve is a hypothetical sunscreen with a similar transmittance shape profile to Sunscreen B, but modified to achieve a *UPF* of 50. One can conceptualize this shift as a person applying sunscreen at a higher areal density than the recommended 2 mg/cm^2^ (which is much higher than that typically applied by consumers [51]). Although the hypothetical dashed curve now represents a *UPF* value of 50, and maintains a broad-spectrum CW of 374 nm, this “new” sunscreen still only blocks 87% of *UVA*, which is considered inadequate relative to the 95% *UVA* blocking criteria used by some countries for sun-protective clothing. As this example demonstrates, the combination of *UPF* and *UVA* blocking may provide more suitable measures for broad-spectrum protection. Numerous jurisdictions, including the European Union, require this combination as an indication of broad-spectrum protection for garments [47].

### 4.5. Limitations of This Study

Our small sample size and use of only one brand of commercial organic sunscreen may limit generalizability, although most brands select from the same ingredient list. Our study did not directly test inorganic UV filters, but the transmission curves generated using the BASF sunscreen simulator [59] (Figure 4) show that ZnO and TiO_2_ demonstrate up to 20% transmittance beyond 380 nm. The high concentrations of ZnO and TiO_2_ required to achieve adequate *UVA* protection may result in a sunscreen that can be difficult to apply and have a chalky, cosmetically less acceptable appearance. The method we used to determine the *UPF* of sunscreens had limitations including variability in sunscreen application on the slide and the slide’s differing properties and structure compared to human skin. Challenges posed by in vitro *UPF* testing of sunscreens have been demonstrated in prior studies, and to date, no in vitro *UPF* test for sunscreens has been approved due to difficulty in generating reproducible and reliable results from lab to lab [72,73].

The *SPF* and *UPF* measurements were made under controlled laboratory conditions. These do not account for the real-world photodegradation of sunscreen UV filters, or the changes in performance due to the known decrease in areal density of the sunscreens as a consequence of friction, sweating, or water exposure [74,75]. In contrast, real-world performance of textiles is likely superior in most situations. In our study, fabrics were taped directly to the skin instead of suspended above the skin. On-skin testing has been reported to drastically reduce *SPF* in fabrics due to the incident UVR passing directly through the open parts of the fabric structure [76,77]. Although the *SPF* of the fabrics tested may represent a “worst-case” scenario, all *SPF*s exceeded 60.

## 5. Conclusions

In our study, we demonstrate that the four tested fabrics provided superior UVB, *UVA*, and overall broad-spectrum protection when compared to two commercial sunscreens. These data, coupled with mounting concerns regarding sunscreen ingredients and excipients (including biological impacts [78], potential environmental harms [79], and difficulty to apply correctly [51]), underscore that clothing should be considered the cornerstone of UV protection. Nevertheless, sunscreen remains an important modality for UV protection, especially on areas of the body such as the face and hands were where clothing may be impractical. In the future, the most effective and widely adopted strategies will likely incorporate both photoprotective clothing and sunscreens with absorbances extending into the visible light range.

Photoprotection modalities, and their regulatory labeling, are imperfect and must evolve with our understanding of the mutagenic potential of solar radiation beyond that of UVB: erythema is not the only biologically relevant endpoint and may not be the endpoint most closely associated with carcinogenesis. To provide comprehensive protection from skin cancer, photo-ageing, and hyperpigmentation, strategies that impede the entire spectrum of potentially damaging solar light are necessary. A better future metric of photoprotection might simultaneously assess total transmittance across the entire spectrum of radiation and then mathematically adjust for the known rate of mutations generated at each wavelength.

## Figures and Tables

**Table 1:** Specifications of tested fabrics (**A**) and commercial sunscreens (**B**). FD = fully drawn, DTY = drawn textured yarn, D = denier *, f = filaments. All knit fabrics are 28 gauge.

**(A) Fabrics**			
**Fabric**	**Yarn(s)**	**Areal Density** **(g/m^2^)**	**Thickness** **(mm)**
Nylon Woven	70D(48f) × 160D(144f)	107	0.36
Polyester Pique Knit	75D(72f) × 75D(36f) FD	180	0.74
Polyester Interlock Knit	50D/72f FD DTY	90	0.48
Polyester Interlock Knit w/TiO_2_ dot print at 30% surface coverage	50D/72f FD DTY	95	0.46
**(B) Commercial Sunscreens**			
**Sunscreen**	**Labeled *SPF***	**Active Ingredients**	
Sunscreen A	30 Broad-spectrum	avobenzone 3%, homosalate 8%, octisalate 4.5%, octocrylene 6%	
Sunscreen B	50 Broad-spectrum	avobenzone 3%, homosalate 10%, octisalate 4.5%, octocrylene 8%	

* Denier is a measure of linear density, an indicator of yarn or filament size. More specifically, denier is the weight in grams of 9000 m of yarn or filament.

**Table 2:** Measured *UPF*, *SPF*, Critical Wavelength, *UVA* and UVB blocking capabilities of studied fabrics and sunscreens. ** Each fabric *SPF* is the average of measurements across three subjects of Fitzpatrick skin types I, II, and III to maximize the human variation studied. Each *UPF* quantity is an average of 5 unique measurements per textile and 10 unique measurements per sunscreen at each of the two concentrations.

Photoprotective Modality	*SPF*	*UPF*	Critical Wavelength (nm)	*UVA*- Blocking	UVB-Blocking
Polyester Pique Knit	77 ± 6	214 ± 21	383	98.49 ± 0.25%	99.76 ± 0.03%
Nylon Woven	60 ± 5	356 ± 41	370	96.14 ± 0.08%	99.93 ± 0.02%
Polyester Interlock Knit	80	492 ± 45	371	97.03 ± 0.27%	99.95%
Polyester Interlock Knit w/TiO_2_ dot print at 30% surface coverage	73 ± 6	649 ± 107	379	98.48 ± 0.28%	99.95%
Sunscreen A (2 mg/cm^2^)	30 ^#^	16 ± 12	371	74.05 ± 10.17%	89.35 ± 8.28%
Sunscreen A (1 mg/cm^2^)	30 ^#^	5.3 ± 3.2	365	54.00 ± 11.33%	76.45 ± 13.46%
Sunscreen B (2 mg/cm^2^)	50 ^#^	31 ± 19	373	82.13 ± 8.71%	94.23 ± 7.16%
Sunscreen B (1 mg/cm^2^)	50 ^#^	14 ± 17	368	65.03 ± 14.64%	84.79 ± 14.42%

** Error indicated as standard deviations; when not indicated, the standard deviation of the measurements is 0. For critical wavelength, the minimum value is reported. ^#^ Commercial sunscreen *SPF*s were taken from the package label.

**Table 3:** UV-protective measurements of textiles from the literature [66] *.

Textile Fabrics	*UPF*	Critical Wavelength (nm)	*UVA* Blocking	UVB Blocking
White Cotton	9	389	91.7%	89.9%
Dark Grey Cotton	98	389	98.8%	99.1%
Denim	2000	389	100%	100%
Polyester	721	387	99.0%	100%

* *UPF* and critical wavelength shown are the values reported in Coyne et al., the *UVA* and UVB blocking is calculated from the digitized average of the measurements.

**Table 4:** Data from Shifting Sunscreen B Transmittance Curve to Simulate *UPF* = 50.

Sunscreen	*UPF*	*SPF*	Critical Wavelength (nm)	*UVA* Blocking	UVB Blocking
Sunscreen B (2 mg/cm^2^)	31 ± 19	50 ^#^	373	82.13 ± 8.71%	94.23 ± 7.16%
Shifted Sunscreen B	50 ± 30	-	374	86.70 ± 5.66%	96.75 ± 4.03%

^#^ Commercial sunscreen *SPF*s were taken from the package label.

## Data Availability

The original data presented in this study are available in the text and supplementary figures. Figure 5 was generated using data available from Coyne et al. [71]. Figure 6 was generated using the publicly available BASF sunscreen simulator: https://sunscreensimulator.basf.com/Sunscreen_Simulator [59].

## Supplementary Materials

The following supporting information can be downloaded at: https://www.mdpi.com/article/10.3390/cancers14030542/s1, Table S1: Inclusion and exclusion criteria of subjects undergoing in vivo *SPF* testing of fabrics. Table S2: Characteristics of the individuals undergoing in vivo *SPF* testing of fabrics.
