# Supplementary material for: Slip versus Slop: A Head-to-Head Comparison of UV-Protective Clothing to Sunscreen
Source: Cancers (Basel). 2022 Jan 21;14(3):542. doi: 10.3390/cancers14030542 (PMC8833350; doi:10.3390/cancers14030542)
Supplement: Supplementary file 1 [file cancers-14-00542-s001.zip › cancers-1505679-supplementary.pdf]

Supplementary Materials

Slip versus Slop: A Head-to-Head Comparison of UV-Protective Clothing to Sunscreen

Elizabeth G. Berry [1,*], Joshua Bezecny [2], Michael Acton [3], Taylor P. Sulmonetti [4], David M. Anderson [4], Haskell W. Beckham [5], Rebecca A. Durr [5], Takahiro Chiba [5], Jennifer Beem [5], Douglas E. Brash [6], Rajan Kulkarni [1,7], Pamela B. Cassidy [1] and Sancy A. Leachman [1]

[1] Department of Dermatology, Oregon Health & Science University, Portland, OR 97239, USA; kulkarnr@ohsu.edu (R.K.); cassidyp@ohsu.edu (P.B.C.); leachmas@ohsu.edu (S.A.L.)
[2] College of Osteopathic Medicine of the Pacific, Western University of Health Sciences, Lebanon, OR 97355, USA; joshua.bezecny@westernu.edu
[3] Exponent, Inc., Natick, MA 01760, USA; macton@exponent.com
[4] Exponent, Inc., Atlanta, GA 30326, USA; tsulmonetti@exponent.com (T.P.S.); danderson@exponent.com (D.M.A.)
[5] Columbia Sportswear Company, Portland, OR 97229, USA; HBeckham@columbia.com (H.W.B.); rdurr@mit.edu (R.A.D.); taka.chiba@columbia.com (T.C.); Jennifer.Beem@columbia.com (J.B.)
[6] Departments of Therapeutic Radiology and Dermatology, Yale University, New Haven, CT 06520, USA; douglas.brash@yale.edu
[7] Portland Veterans Administration Medical Center, Portland, OR 97239, USA
* Correspondence: berryel@ohsu.edu; Tel.: +1-(503)-418-3376

Table S1. Inclusion and exclusion criteria of subjects undergoing in vivo SPF testing of fabrics.

Inclusion Criteria	Exclusion Criteria
1. Individuals between eighteen and seventy years old<br>2. Individuals free of any dermatological or systemic disorder which would interfere with the results<br>3. Individuals free of any acute or chronic disease that might interfere with or increase the risk of study participation<br>4. Individuals with untanned skin on the test area and with Fitzpatrick Skin Type I, II, III and/or ITA° value >28° by colorimetric method<br>5. Individuals with no uneven skin tones, pigmentation, scars, other irregularities or hair in test site areas that would interfere with SPF determination<br>6. Individuals who complete a preliminary, mandatory medical history form and are in general good health.<br>7. Individuals who will read, understand and sign an informed consent document relating to the specific type of study they are subscribing<br>8. Individuals able to cooperate with the investigator and research staff, be willing to have test materials applied according to the protocol, and complete the full course of the study<br>9. Individuals willing to refrain from using any sunscreen products, sunbathing, or tanning bed use, 24 hours prior to study initiation and the entire duration of the study<br>10. Individuals with excessive hair on their back who are willing to have hair removed by AMA technicians prior to commencement of study	1. Individuals under a doctor's care<br>2. Individuals below the age of consent or older than 70 years<br>3. Individuals currently taking any medication (topical or systemic) with photo-sensitizing potential<br>4. Subjects with a history of dermatological conditions (any form of skin cancer, melanoma, lupus, psoriasis, connective tissue disease, diabetes or any disease that would increase risk associated with study participation)<br>5. Individuals diagnosed with chronic skin allergies<br>6. Subjects using anti-inflammatory medication<br>7. Subjects accustomed to using tanning beds<br>8. Subjects having had sun exposure on the back area in the previous four weeks prior to SPF testing<br>9. Subjects having excessive hair in the area of the test<br>10. Individuals with a history of adverse effects upon sun exposure<br>11. Female volunteers who indicate that they are pregnant or lactating

Table S2. Characteristics of the individuals undergoing in vivo SPF testing of fabrics

Textile	Subjects Enrolled	Testing Dates	Age Range (years)	Sex	Race/Ethnicity	Fitzpatrick Photo-types
**Nylon Woven**	3	9/11/2019<br>10/9/2019<br>1/15/2020	36-47	2M, 1F	3 Caucasian	I, II, III
**Polyester Pique Knit**	3	9/11/2019<br>10/9/2019<br>1/15/2020	36-47	1M, 2F	2 Caucasian, 1 Hispanic	I, II, III
**Polyester Interlock Knit**	3	9/11/2019<br>10/9/2019<br>1/15/2020	36-47	2M, 1F	3 Caucasian	I, II, III
**Polyester Interlock Knit with TiO_2**	3	9/11/2019<br>1/14/2020<br>1/15/2020	42-64	1M, 2F	2 Caucasian, 1 Hispanic	I, II, III
